# Phosphorus—Sexual Weakness; Arsenic—Piles; Sulphur—Post-partum Melancholia; Sulphur—Piles; Phos.—Deafness

**Published:** 1881-04

**Authors:** E. W. Berridge

**Affiliations:** London


### CLINICAL CASES.

BY E. W. BERRIDGE, M. D., LONDON.

*Phosphorous*—Sexual Weakness; *Arsenic*—Piles; *Sulphur*—Post-partum—Melancholia; *Sulphur* in Prolapsus and Hæmorrhoids; *Phosphorous* in Deafness.

PHOSPHORUS IN SEXUAL WEAKNESS.—1877, July 17th, Mr. —— has been married two months. For two years has had emissions, at first about every two months, but since marriage very often; they occur during sleep, and are accompanied by amorous dreams. Does not feel any escape of semen during coition. Erection too short; sexual pleasure slight, and only experienced at the commencement of coition. The greater the desire for coition the greater is the emission some hours after the act.

DIAGNOSIS OF REMEDY.—Emissions after coition: *Baryt.*, *Kali carb.*, *Nat. Mur.*, *Phos.*, *Rhod.*

Emissions with dreams: *Baryta*, *Kali carb.*,*Phos.*, *Rhod.*, (with many others which have not the forgoing symptom.)

Emission without force: *Con.*, *Phos.*

The list is thus reduced to *Phos.*, and as this remedy produces depression as well as exaltation of the sexual functions, I gave him one dose of M.M. (Fincke).

Oct. 12th. Reports that emissions ceased in a week, and did not return till last night, then one occurred soon after coition without intervening sleep; on waking felt miserable; after coition feels exhausted. *Phos.* M.M. (Fincke) one dose.

Feb. 10th., 1878. Reports that emissions have ceased for three months; sexual power and pleasure normal. Depression of the sexual powers being the *secondary* action of *Phos.*, this cure by a high potency not only shows that the secondary symptoms (to which *Hahnemann at first* attached no importance, though he altered his views *later*) possess diagnostic value, but that Dr. E. M. Hale's so-called "law of dose" is inaccurate.

*Arensic.* IN PILES.—1879, Mrs. —— had a pile on left side of anus, large, with appearance of black veins; frequent ineffectual desire for stool, with nausea and loss of appetite. In the pile there is a dull, aching pain, with occasional burning and shooting upward; relief from lying down, worse from sitting and standing.

Diagnosis of Remedy.—Piles better by lying down, *Amm.*, *Carb.*, *Arsen.*

Piles worse sitting, *Arsen.*, (and many others.).

The Materia Medica shows that *Arsen.* had also the burning shooting in piles; and one dose of C. M. (Skinner's F. C.) was given in the evening. Next day she was very much better; bowels acted naturally and she was soon well. Some months afterward there was a return of the symptoms; one dose of *Arsen.*, M.M. (Fincke) cured promptly. She has now had no return for many months.

*Sulphur* in Post-partum Melancholia.—June 26th, 1878.—Mrs. —— was delivered of her third child three months ago; was much worried, and had extra labor a month before the event, and felt worried afterward. After delivery had strange fancies, e. g., that the nurse's face was powdered; much excitement; milk ceased in fourteen days; was sleepless; felt that her head was enlarged, and that she was too tall or too short; with intolerance of noise. She took from an allopath, *Sal Volatile*, *Valeria*, *Lavender*, *Chloroform*, and *Bromide of Potass.* This treatment removed the intolerance of noise, but left the following symptoms, which the doctor says will disappear in the course of time; but with this comforting assurance she is not satisfied.

Present state: feels indifferent to things, even to her children; indifferent to pleasant things, but alive to disagreeable; sits with out thinking or doing anything; hopeless of recovery; desire for solitude; more cheerful after meals; intense longing to get out of her mental state, of which she is quite conscious; weeps at times, but it does not relieve her; forgets the word she is going to use, and what she is about to do; desire to die; restlessness, only in the house as if she must walk about; confusion of head if she thinks much about her household duties. Ever since confinement, feeling of want of faith in Providence; took last dose of the *Bromide* and *Chloroform* this morning.

DIAGNOSIS OF REMEDY.—Taking as the starting point the "more, striking, singular, uncommon and peculiar (characteristic)" symptom of the relief of the mental symptoms from food, I found that *Sulph.* has "weeping relieved by eating;" and as it corresponded well with the other symptoms, I gave one dose of *Sulph.*, D. M. (Skinner's F. C.).

28th. Reports not so well; increased restlessness, and greater aversion to do any work, but has slept as well since leaving off the sedative as before. This latter feature convinced me that the increase of symptoms was an aggravation from the *Sulph.*, and not the effect of stopping the allopathic treatment. No medicine.

July 5th. Reports that she feels much better; not so restless. No medicine.

10th. Has had more restlessness and depondency, but is much better to-day.

Sept, 30th. Her husband reports that she has remained well ever since.

*Sulphur* IN PROLAPSUS AND HÆMORRHOIDS. —Aug. 24th, 1878. Mrs. —— was confined nine months ago; ever since, at times, she has suffered from piles, with soreness there like a blister, and sometimes burning, relieved by the application of lard. For fourteen days, prolapsus of rectum for about an inch, first noticed when walking, with great soreness and throbbing. For five months at times pain in coccyx like drawing a tooth, felt this on rising from sitting, and a little if she sits long, not when standing or lying.

DIAGNOSIS OF REMEDY.—Dislocated pain in coccyx is found only under *Sulph.*, which also has all the other symptoms. I gave one dose of *Sulph.*, D.M. (Skinner's F.C.).

Sept. 2nd. The prolapsus ceased gradually, and after the 28th. Aug. was gone; the soreness and throbbing better on the 25th., gone on the 26th. ; the coccygeal pain unchanged.

10th. No return of prolapsus, piles, or rectal pains, coccygeal pain better for a week.

28th. Coccyx well for nine or ten days; no return of the other symptoms.

Feb. 19th., 1879. Has remained well.

In the above case the pain attending the prolapsus was relieved before the prolapsus itself, just as when the homœopathic remedy is given for abscess or calculi, the pain is first relieved, and afterward the objective symptoms disappear. The coccygeal pain being of longer duration than the prolapsus took longer to remove. That the oldest symptom, the piles, disappeared before the pain in the coccyx, does not contradict Hahnemann's teaching, because, when the prolapsus was cured they would necessarily improve at once from their interdependence.

*Phosph.* IN DEAFNESS.—March 7th., 1879. As the result of a cold, I had been deaf in the left ear for ten days; the ear felt stopped up, with singing therein; the watch applied to mastoid processes was heard equally well in both sides; but when held before the ears there was a marked difference, both in the distance at which it was audible, and also in the degree of clearness with which it could be heard at the same distance from either ear. At first the deafness was only in the morning, on and a little after waking, I took *Silicea* C.M. (Fincke), but without result. Then the deafness continued the same all day; I then took *Arg. Nit.*, which only slightly relieved the singing for a time. For the last two days, when exposed to the noise of traffic in the street—the louder the traffic, the more marked was the symptom—I heard bells chiming, always with the left ear only. The bells were heard when riding in a tram car or train, or even when the car was still, if the street happened to be noisy and also from walking where there was much noise from traffic. The other symptoms were worse. I now took *Amm. Carb.* 200 (one dose), and continued with 2300 (Jenichen).

8th. After the first dose, taken at 8.30 P. M., I was a little better, the bells not quite so loud, and to-day I could hear watch at a greater distance than before.

13th. Continued the *Amm. Carb.* with improvement till the 10th, after which I took no more because an aggravation of the deafness had occurred, though on that day I heard no bells while in the train. The symptoms now increased again; the bells were more troublesome than before; for the last few days *deafness was momentarily relieved by boring finger in left ear, or by pressing on it externally;* at times slight deafness also in right ear, relieved by boring with finger. As the symptoms were increasing, and even extending to the other ear, in spite of leaving off the *Amm. Carb.*, which seemed to have first relieved and then aggravated, I concluded that the last remedy had now done all it could, and that another must be selected.

The italicized symptoms, which were the latest, and so (*caeteris paribus*) the most characteristic pointed to *Phosph.*, of which I took one dose C.M. (F. C., Skinner) at 9.40 A. M. At 10.55 A. M. there was a *remarkable* change. I could hear the watch at a greater distance than since my deafness, and much more distinctly. At 5.15 P. M. heard the bells but less; ear felt more natural; less singing; hearing still improving. In evening bells nearly gone; hearing nearly natural.

14th. On waking and a little afterward, increased deafness and singing; this soon subsided, and by 10.10 A. M. there was scarcely any singing, and hearing was nearly natural; no bells all day.

15th. Deafness and singing on and a little after waking, as before; soon improved. In afternoon there great noise of traffic, heard the bells once.

16th. No aggravation on waking, left ear still a little deaf.

18th. The old symptoms on waking returned; at 10 A. M. rather more deafness of left ear; no more bells.

19th. No morning aggravation; hearing nearly natural.

22d. Watch sounds a little sharper when applied to right ear than when applied to left; no other symptom.

29th. Perfeclly well.

June 26th., 1881. Have remained quite free from these symptoms to this day, and I am thankful that the fate of Irving's "Matthias" is no longer mine, thanks to homœopathy.

COMMENTS. (1) The earliest symptom, the deafness in the morning *on and after waking*, which was the first to appear was also the last to disappear, as Hahnemann teaches.

(2) The aggravation *in a noise* is peculiar, and, as far as I know, is new to the Materia Medica; verifications are solicited.

(3) The first three prescriptions failed because a complete picture of the case had not been obtained; in these cases where a partially homœopathic remedy has been given, new symptoms often arise; these are of the greatest importance in the selection of the *simillimum;* they are the voice of Nature pointing out the physincian's error, and how he may rectify it.

(4) The slight aggravation *after* the improvement which Hahnemann points out, was manifest in this case; under such circumstances the remedy should always be allowed to act without change or repetition.

